# Alzheimer's disease disrupts alpha and beta-band resting-state oscillatory network connectivity

**DOI:** 10.1016/j.clinph.2017.04.018

**Published:** 2017-11

**Authors:** Loes Koelewijn, Aline Bompas, Andrea Tales, Matthew J. Brookes, Suresh D. Muthukumaraswamy, Antony Bayer, Krish D. Singh

**Affiliations:** a CUBRIC, School of Psychology, Cardiff University, Maindy Road, Cardiff, UK; b Department of Psychology, College of Human and Health Sciences, Swansea University, Swansea, UK; c Sir Peter Mansfield Magnetic Resonance Centre, School of Physics and Astronomy, University of Nottingham, Nottingham, UK; d Schools of Pharmacy and Psychology, University of Auckland, Auckland, New Zealand; e School of Medicine, Cardiff University, University Hospital Llandough, Cardiff, UK

**Keywords:** Alzheimer’s disease, Magnetoencephalography, Neural oscillations, Functional connectivity, Default-mode network, Resting state

## Abstract

**Objective:**

Neuroimaging studies in Alzheimer’s disease (AD) yield conflicting results due to selective investigation. We conducted a comprehensive magnetoencephalography study of connectivity changes in AD and healthy ageing in the resting-state.

**Methods:**

We performed a whole-brain, source-space assessment of oscillatory neural signalling in multiple frequencies comparing AD patients, elderly and young controls. We compared eyes-open and closed group oscillatory envelope activity in networks obtained through temporal independent component analysis, and calculated whole-brain node-based amplitude and phase connectivity.

**Results:**

In bilateral parietotemporal areas, oscillatory envelope amplitude increased with healthy ageing, whereas both local amplitude and node-to-global connectivity decreased with AD. AD-related decreases were spatially specific and restricted to the alpha and beta bands. A significant proportion of the variance in areas of peak group difference was explained by cognitive integrity, in addition to group. None of the groups differed in phase connectivity. Results were highly similar for eyes-open and closed resting-state.

**Conclusions:**

These results support the disconnection syndrome hypothesis and suggest that AD shows distinct and unique patterns of disrupted neural functioning, rather than accelerated healthy ageing.

**Significance:**

Whole-brain assessments show that disrupted regional oscillatory envelope amplitude and connectivity in the alpha and beta bands play a key role in AD.

## Highlights

• Whole-brain resting-state MEG shows decreased connectivity in AD vs. increases in healthy ageing.
• Results unique to parietotemporal areas and beta band in AD, highly similar for eyes open and closed.
• These unbiased results suggest distinct patterns of dysfunction rather than accelerated ageing in AD.

## 1 Introduction

Alzheimer’s disease (AD) is a progressive degenerative disease, affecting memory and other cognitive functions. The neural characteristics of AD are structural changes such as widespread atrophy and deposits of amyloid plaques and tau protein neurofibrillary tangles, particularly in the hippocampus, entorhinal cortex and post-central parietal areas (Lizio et al., 2011).

Non-invasive structural imaging, such as diffusion imaging, mainly shows that in AD, as well as to a lesser degree in mild cognitive impairment (MCI), white matter integrity is reduced compared to elderly controls in a number of cortical and subcortical connections (Rose et al., 2000, Bozzali et al., 2002, Zhang et al., 2009, Acosta-Cabronero et al., 2010, Douaud et al., 2013). In addition to these white matter degeneration patterns, grey matter atrophy is evident, in particular in hippocampal and entorhinal cortex (Baron et al., 2001, Karas et al., 2003), starting in the medial temporal lobes (Smith, 2002). In contrast to these specific patterns in AD, healthy ageing shows a more global degeneration pattern with a frontal emphasis (Madden et al., 2009, Penke et al., 2010, Fjell et al., 2011). These structural patterns therefore support a specific pathological network disconnection in AD and hence challenge the notion that AD presents with a pattern of accelerated or exaggerated ageing. However, structural abnormalities in AD may not be revealed by non-invasive imaging during the earliest stages of disease, particularly when predicting conversion from MCI. It may be that in the early stages functional measures such as fMRI, EEG and MEG are more sensitive.

Resting-state brain function in AD has therefore been intensively studied using functional magnetic resonance imaging (fMRI), where background resting-state networks of coherent spontaneously occurring blood oxygenation level dependent (BOLD) activity can be identified using independent component analysis (ICA) (Beckmann et al., 2005, Damoiseaux et al., 2006). These fMRI studies often focus on the default-mode network (DMN), and generally show a reduction in DMN-wide or local node activity in AD (e.g., Greicius et al., 2004, Liu et al., 2014). However, the BOLD signal reflects an indirect measure of neural activity by measuring oxygenation changes in the blood. The interpretation of the fMRI BOLD signal is hampered by its potential physiological confound, because non-neural factors such as cardiac and respiratory events may also influence local metabolic demand, and thus blood flow and composition, in a regular temporal manner (Murphy et al., 2013). This is particularly problematic for the study of ageing and degenerative diseases because of the potential link to systemic physiological problems.

Furthermore, fMRI is not capable of measuring oscillatory signals at higher frequencies, thought to be crucial for neural communication (Engel et al., 2013). Brain function in AD has therefore also been studied extensively using more direct neurophysiological techniques such as EEG and MEG. A common finding in M/EEG studies in AD is that posterior alpha and beta power decreases compared to elderly controls (EC) or mild cognitive impairment (MCI) (Moretti et al., 2004, Osipova et al., 2005, Hsiao et al., 2013). These decreases correlate with altered cerebral blood flow and mini-mental state examination (MMSE) scores (Lizio et al., 2011, Babiloni et al., 2015), as well as with occipital, but also temporal, grey matter density (Babiloni et al., 2015). In healthy ageing, alpha oscillations also decrease in occipital areas, as well as in parietal and temporal areas (Babiloni et al., 2006a). Oscillatory measures in MCI fall in between AD and EC, particularly in the alpha band (Koenig et al., 2005, Lizio et al., 2011). These findings, together with the pattern of disrupted cognitive functions, therefore suggest that changes in brain and behaviour in AD could indeed be described as exaggerated or accelerated ageing.

However, not all M/EEG findings fit with the concept of accelerated ageing in AD. A second common finding in M/EEG studies is referred to as occipital alpha ‘slowing’, a general decrease in individual alpha-band mean peak frequency (IAF) in AD (Poza et al., 2007, Montez et al., 2009, Garcés et al., 2014, Babiloni et al., 2015). Alpha slowing may predict progression of MCI to AD, but is not consistently observed in healthy ageing (Babiloni et al., 2006a, Cantero et al., 2009). In line with alpha slowing, power in the delta and theta bands has been found to be increased in AD compared to EC or MCI (Moretti et al., 2004, Osipova et al., 2005, Hsiao et al., 2013), and delta and theta power in MCI are generally in between healthy ageing and AD (Babiloni et al., 2006b, Lizio et al., 2011). However, this lower-frequency pattern is not consistently reported or observed. Studies investigating correlations between oscillatory signals in the phase domain have also found an inconsistent pattern of region-specific decreases and increases that vary over frequency bands in both MCI and healthy ageing (Cantero et al., 2009, Buldú et al., 2011) as well as in AD (Stam et al., 2005, Stam et al., 2006, de Haan et al., 2012).

Clearly, there is considerable variability and complexity in M/EEG findings of oscillatory amplitude and frequency. Attempts to reconcile this complexity have claimed that it is more appropriate to study AD in terms of brain network organisation (Stam, 2014), and generally find increased connectivity with healthy ageing (Buldú et al., 2011) as opposed to disconnection in AD (Lizio et al., 2011). Studies employing Graph Theory Analysis show a loss of ‘small worldness’ in MCI and AD, i.e., an increase in randomness and thus a less organised system (Buldú et al., 2011, Babiloni et al., 2016), although results using ‘modularity’ (i.e., the amount of clustering, another measure of organisation) vary over frequency bands (de Haan et al., 2012). Fronto-parietal and fronto-temporal connections appear most affected (Babiloni et al., 2016), and imaging studies using various modalities converge on a general between-lobe or module (long-distance) decrease in functional connectivity in AD, often expressed as a loss of hubs (de Haan et al., 2012, Tijms et al., 2013). Within this framework, AD has therefore been proposed to be a ‘disconnection syndrome’ (Delbeuck et al., 2003, Pineda-Pardo et al., 2014, Stam, 2014).

All of the MEG/EEG studies reviewed here are limited in either the chosen frequency band(s), the studied brain area(s) or network(s), the measure of neural activity or connectivity, or they did not localise signal sources to the brain. Furthermore, these studies typically recorded the MEG data with either eyes open or closed, the effect of which has never been compared. All of these factors may influence the results (Stam, 2014), potentially resulting in conflicting or partial information. Here we provide a comprehensive study of whole-brain connectivity changes in AD compared to EC in the MEG resting state, limiting any bias in terms of *a priori* decisions as much as possible. We made use of recent developments that allow us to study the electrophysiology of background neural networks non-invasively (de Pasquale et al., 2010, Brookes et al., 2011). Furthermore, we contrasted changes in AD to changes in healthy ageing by including a young control group (YC). Finally, we investigated the effect of keeping eyes open and closed during the MEG resting-state recording.

## 2 Methods

### 2.1 Subjects

Twenty-one patients diagnosed with early stage probable Alzheimer’s disease (AD) (McKhann et al., 1984) were recruited through clinics. We further recruited twenty-three aged-matched healthy elderly controls (EC), spouses of patients or via our local Community Panel, and sixteen healthy young controls (YC). Inclusion criteria for all AD and elderly subjects were a Mini-Mental State Examination (MMSE) score of 19 or more (Mean ± SD, range; AD: 22.4 ± 2.5, 19–27; EC: 29.3 ± 0.9, 27–30), ability to give informed consent, no MRI contraindications, ability to cope with the demands of the study, no systematic illness or drugs likely to impact significantly on cognition (or if on cholinesterase inhibitor, to have been on a stable dose for at least 8 weeks), adequate vision, and recent MMSE result available. For probable AD, further inclusion criteria were that dementia has been established by clinical and cognitive assessment (ACE-R), cognitive impairments are progressive and include episodic memory deficit and deficit in at least one other area of cognition, and absence of other conditions that explain the dementia syndrome, including any evidence of significant cerebrovascular disease on a previous CT scan. Healthy elderly controls were further required to have no evidence of cognitive impairment (MMSE ≥ 27, data on 2 controls were missing), no concern about their memory or cognitive performance and had not approached their GP (or memory services) with any concerns about such function. One patient could not perform the scans and four young controls did not perform the required MEG paradigm for the analyses in this paper. To increase the number of datasets in the YC group, datasets from ten young controls performing the same paradigm were added from a different study acquired at CUBRIC (the ‘100 Brains’ study). All YC were required to have no current neurological or neuropsychiatric episode, or other mental health issues. All participants gave informed consent and all procedures were approved by the local Ethics Committee.

Datasets were assessed for quality and head motion. As it could be expected that elderly subjects have greater difficulty staying still than younger subjects, we allowed a 10 mm maximum head motion for all datasets to avoid excessive loss of data (e.g., Stam et al., 2006). One young control had excessive amounts of bad data (>3 SD from the whole-group mean) and four AD patients and two elderly controls had head motion exceeding 10 mm. Data from these subjects were excluded from analysis. All analysis is therefore based on *N* = 16 AD patients (6F/10 M, age 67–89, mean 77.5 years), *N* = 21 EC (13F/8 M, age 67–87, mean 73.8 years) and *N* = 21 YC (11F/10 M, age 20–56, mean 28.8 years), with a total *N* = 58. On average, the AD patients were slightly older than the EC subjects, but this was not statistically different (*t*(35) = 1.74, *p* = 0.09). Although attempts were made to match years of education, there was a small difference where AD patients had fewer years of education than controls (AD: 9–19, mean 12.4; EC: 9–24, mean 15.3, 1 missing; *t*(34) = 2.26, *p* = 0.03). The three groups did not significantly differ in number of epochs included in the analysis (mean: AD = 164, EC = 165, YC = 166, F(2,55)=1.21, *p* = 0.31), nor head motion (mean ± SD: AD = 0.46 ± 0.28 cm, EC = 0.38 ± 0.29 cm, YC = 0.25 ± 0.25 cm, F(2,55)=2.55, *p* = 0.09).

### 2.2 Data acquisition

Whole-head MEG recordings were made using a 275-channel CTF radial gradiometer system. An additional 29 reference channels were recorded for noise cancellation purposes and the primary sensors were analysed as synthetic third-order gradiometers (Vrba and Robinson, 2001). Two or three of the 275 channels were turned off due to excessive sensor noise (depending on time of acquisition). Subjects were seated upright in the magnetically shielded room. To achieve MRI/MEG co-registration, fiduciary markers were placed at fixed distances from three anatomical landmarks identifiable in the subject's anatomical MRI, and their locations were verified afterwards using high-resolution digital photographs. Head localisation was performed before and after each recording, and a trigger was sent to the acquisition computer at relevant stimulus events.

For the analysis reported in this paper, we required a ‘resting-state’ recording in which we asked subjects to rest and sit comfortably in the MEG chair, while their head was supported with a chin rest. Subjects heard a voice through earphones, which informed them with a single word to either close their eyes (‘close’) or open them (‘open’) in alternating fashion. Each alternation lasted 15 s, with 12 alternations of each state, yielding 24 × 15 s repeats, taking 6 min in total.

In the ‘eyes-open’ part of the experiment, a subset of subjects (14 AD, 11 EC, 11 YC) viewed a red dot superimposed on a grating. The remaining subjects viewed a red dot superimposed on a uniform grey background. All subjects were asked to focus their eyes on the red dot. Displays were generated in MATLAB® (The MathWorks, Inc.), using the Psychophysics Toolbox extensions (Brainard, 1997, Pelli, 1997, Kleiner et al., 2007), and were presented on a Mitsubishi Diamond Pro 2070 monitor (1024 × 768 pixel resolution, 100 Hz refresh rate).

All datasets were either acquired at or down-sampled to 600 Hz, and filtered with a 1-Hz high-pass and a 150-Hz low-pass filter. The datasets were then segmented into 2-s epochs excluding the first second after each change in eye state. The data were visually inspected and epochs with major artefacts such as head movements or large muscle contractions were excluded from subsequent analysis. Finally, the datasets were split into two separate datasets per subject containing eyes open and closed epochs, respectively, yielding 116 datasets entering the group analysis.

Subjects further underwent an MRI session in which a T1-weighted 1-mm anatomical scan was acquired, using an inversion recovery spoiled gradient echo acquisition. For two patients, the MRI scan could not be acquired or was unusable due to technical difficulties. In these cases, for the purpose of MEG-MRI co-registration, we replaced the missing scan with an MRI scan of another patient of the same gender.

### 2.3 Resting-state independent component network analysis

We analysed oscillatory resting networks using the methodology described by Brookes and colleagues (Brookes et al., 2011, Hall et al., 2013), similar to our previous work (Muthukumaraswamy et al., 2013, Koelewijn et al., 2015). Using the pre-processed data, beamformer weights were computed on an 8-mm grid for each subject and frequency band. Beamformer time courses were then generated at every voxel and normalised by an estimate of the projected noise amplitude at that voxel (Hall et al., 2013). The Hilbert transform was applied to each voxel time course, and the absolute value was computed to generate an amplitude envelope of the oscillatory signals in each frequency band. The data at each voxel were down-sampled to an effective sampling rate of 1 Hz (Luckhoo et al., 2012), and transformed to the MNI template brain using FLIRT in FSL. Data from all subjects and both eye states were then concatenated in the time dimension across subjects.

Temporal independent component analysis (ICA) was applied to the concatenated datasets using the fast ICA (research.ics.tkk.fi/ica/fastica) algorithm. We applied ICA separately for six bands commonly used in previous literature: 1–4 Hz (delta), 4–8 Hz (theta), 8–13 Hz (alpha), 13–30 Hz (beta), 30–50 Hz (gamma), and 50–90 Hz (high gamma) (Brookes et al., 2011, Muthukumaraswamy et al., 2013). Pre-whitening was applied to reduce the dimensionality of the source-space Hilbert envelope signals to 20 principal components before ICA (Hyvärinen and Oja, 2000, Brookes et al., 2011, Hall et al., 2013). Fifteen independent components were derived per band.

We determined, *a priori*, to focus only on clear-cut bilateral or multi-focus unilateral networks that our lab and others have consistently found using this ICA approach in a particular frequency band (Brookes et al., 2011, Muthukumaraswamy et al., 2013, Koelewijn et al., 2015) and which correspond well with well-known functional networks from fMRI research (Brookes et al., 2011). This approach served to minimise multiple comparisons across networks and frequency bands. The networks that satisfied these criteria were the sensorimotor network, the visual network, and the left and right parietofrontal networks. Furthermore, to avoid frequency band as an additional factor in the analysis, we intended to select the components within a single frequency band in which all four networks were clearly present as separate components. Based on previous research, we hypothesised that the four networks would all be clearly present only in the 13–30 Hz beta band (Brookes et al., 2011, Brookes et al., 2012).

The result of the ICA analysis is a set of 3-dimensional (‘volumetric’) spatial maps reflecting temporally correlated oscillatory envelopes. In order to compare a measure of activity within these components of interest between groups, we used a region-of-interest (ROI) approach to obtain a single measure of network activity per dataset. To achieve this, we first computed a volumetric (voxel-based) image of the time-series standard deviation (SD) of the 1-Hz beta-band envelope for each dataset. We then thresholded the absolute volumetric activity map of each component of interest at 0.5 to yield a binary mask. This mask was then applied to each dataset’s SD image (subject and eye state) separately to extract the mean SD over the relevant voxels within each network, yielding a single ‘summary’ measure per dataset per network. The component time-series SD can be interpreted as a measure of activity in a network (Muthukumaraswamy et al., 2013), or variability thereof (Koelewijn et al., 2015). We calculated the SD, because the components were de-meaned, meaning that the average would be close to zero and is thus uninformative.

The beamformer weights generated by the ICA network calculations were normalised by an estimate of noise projected through the beamformer weights (Hillebrand et al., 2012). Any systematic differences in this normalisation between the groups could potentially bias our ICA component results. To assess whether there was any such bias, we therefore conducted a statistical comparison of the vector magnitude of the weights at each location of the source-reconstructed images. These images were first spatially normalised to the MNI template using FSL FLIRT with an affine transform. We then applied the same ROI analysis to these images using the same respective component masks as we applied in the component amplitude SD analysis.

The temporal ICA analysis is conducted with the assumption that known functional resting-state networks are spatially stationary across individuals and thus groups. To verify whether this assumption was valid, we further conducted a temporal ICA, identical to the method described previously, on each group separately, for the beta band only.

### 2.4 Whole-brain voxel-based analysis on envelope SD

As the network masks were quite large, there is a degree of overlap between them. As we take a crude global average measure within each network, it is possible that a single cluster underlies each network’s group difference. To identify hot-spots of group differences across the brain we additionally performed a whole-brain voxel-based group comparison on the Hilbert envelope SD of the 8 mm voxels on the 1 mm MNI template brain (randomised two-sample t test corrected for multiple comparisons across voxels, 2-tailed, *p* < 0.05, 1000 permutations). We performed this analysis for each frequency band. We used a conservative approach and limited our analyses to the cerebral cortex.

### 2.5 Node-based connectivity analysis

The ICA analysis allowed us to investigate global group differences within existing functional networks of areas that are consistently temporally active together within a frequency band. However, this does not tell us how different areas across the brain correlate and whether the strength of this correlation is modified by disease. To investigate this question, we conducted a whole-brain connectivity analysis, with the number of data points reduced for computational demands and statistical power. We used FieldTrip version 20150610 (Oostenveld et al., 2011) to perform source localisation on the datasets identically preprocessed to the ICA analysis. Source localization was performed using a SAM beamformer on a 6-mm grid, using a localspheres forward model, where the covariance matrix was constructed per frequency band for each of the following bands: δ 1–4, θ 4–8, α 8–13, β 13–30, γ1 30–50, and γ2 50–90 Hz. For each band, the beamformer weights were normalized using a vector norm (Hillebrand et al., 2012). After constructing the beamformer weights on the 6-mm grid, the data was normalised to the MNI template, and reduced to 90 nodes, with one node per region of the Automatic Anatomical Labelling atlas (Tzourio-Mazoyer et al., 2002). Per dataset, the selection of one node per AAL region was achieved by performing a frequency analysis on all virtual channels (nodes on the 6-mm grid) within the AAL region, and the virtual channel with the greatest power was selected to represent that region.

We calculated two measures of correlation across nodes. We calculated connectivity between the amplitudes of oscillatory envelopes, as well as between their phases. These two types of coupling are thought to reflect different roles in brain function, with phase coupling playing a role in neural communication on more distant and faster temporal scales than envelope correlations, which may be more involved in preparing neural populations for input (Engel et al., 2013). Both coupling mechanisms are likely to play a role in establishing functional networks.

To calculate the Orthogonalised Amplitude Correlation (OAC), the Hilbert envelope was taken per trial, then the data were trimmed avoiding the first and last 100 samples, and downsampled by a factor of 4. The Hilbert envelopes were then orthogonalised to avoid spurious correlations (Hipp et al., 2012). OAC adjacency matrices were constructed by correlating the envelope amplitudes of each node to each other node, generating a 90 ∗ 90 matrix per frequency band per dataset. To calculate Phase Lag Index (PLI), we used a Fourier decomposition using a Hanning window, and then constructed 90 ∗ 90 adjacency matrices by calculating the debiased weighted PLI across nodes (Stam et al., 2007).

For both OAC and PLI independently, we performed statistical group comparisons on the sum of each respective measure per node (‘strength’ in graph theoretical analysis, a measure of ‘connectedness’ of each node across all nodes in the brain). We compared these measures for EC versus YC to assess the effect of healthy ageing, and for EC versus AD to assess the effect of Alzheimer’s disease, using two-sample t tests (randomised by 1000 permutations, *p* < 0.05 corrected for multiple comparisons across nodes). Finally, we mapped the results for each group comparison back onto the brain by assigning the t value of each significant node onto each respective AAL region for visualisation.

### 2.6 Partial least squares analysis on MMSE and other factors

To assess the relative contribution of a number of factors on the beta-band source-space results obtained for the EC and AD groups, we performed a partial least squares (PLS) regression (randomise-controlled using 10,000 permutations) with as predictor variables all variables that showed a difference, or trend for a difference, between the EC and AD groups. We performed the PLS regression using a randomised adaptation of the ‘plsregress’ function from Matlab’s statistical toolbox. We ran this separately for the voxel-based SD and AAL-OAC measures. For both measures, we calculated individual values at the left and right region (voxel, or AAL node, for the respective analyses) where the group difference of EC vs. AD was maximal, and then averaged the outcome over eyes open and closed. Missing values were excluded in this analysis. Therefore, data was entered for *N* = 16 AD patients and *N* = 18 EC.

### 2.7 Sensor-space analysis

The MEG ICA approach is relatively novel and has to our knowledge not yet been applied in Alzheimer’s disease or the elderly population. Our novel findings can therefore be difficult to interpret or verify. To increase reliability and interpretability of our ICA findings, we replicated a number of consistent electrophysiological findings in the AD literature in the same datasets we applied our ICA analysis to, using a sensor-space analysis (Supplementary Material; Appendix A).

## 3 Results

### 3.1 Resting-state independent component network analysis

**Fig. 1:**
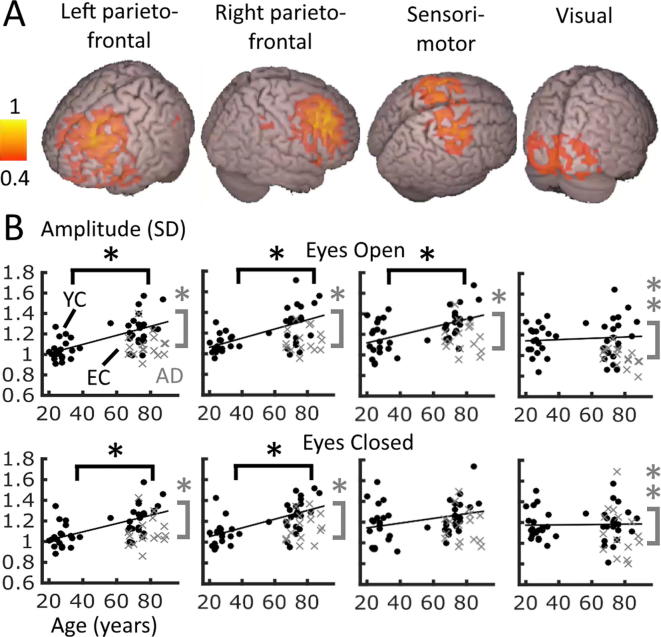
Temporal oscillatory ICA network results generated on all data (all groups and both eye states). (A) Spatial maps of four consistent network components in the beta band transformed to MNI space. Images show absolute ICA weights (in A.U.) thresholded above 0.4, scaled to a maximum of 1. (B) SDs of beta-band oscillatory envelopes averaged within the spatial boundaries of each network, plotted over age per network and eye state. Black circles: control data, grey crosses: AD. AD = Alzheimer’s disease patients, EC = Elderly controls, YC = Young controls. Statistics indicate ANOVA post hoc t test results: ^*^p < 0.05 for the relevant 2-group comparison (EC > YC, black; AD < EC, grey), ^**^p < 0.05 for AD < EC&YC, Bonferroni corrected. Per plot, a least-squares line is fitted for all relevant control data over age for illustration of the group difference direction only.

**Fig. 2:**
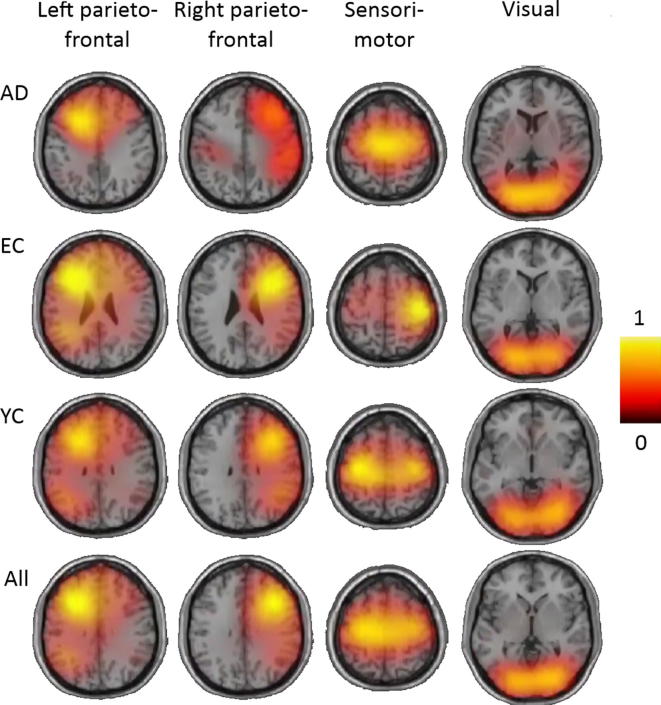
Per-group comparable ICA spatial network components transformed to MNI space. Each image displays an axial slice positioned at component maximum. Each ICA was performed on eyes-open and closed data combined. AD = Alzheimer’s disease patients, EC = Elderly controls, YC = Young controls. The bottom row (All) shows the original ICA analysis performed on all groups together equivalent to Fig. 1. Images show absolute ICA weights (in A.U.), scaled to a maximum of 1.

**Supplementary Fig. S1:**
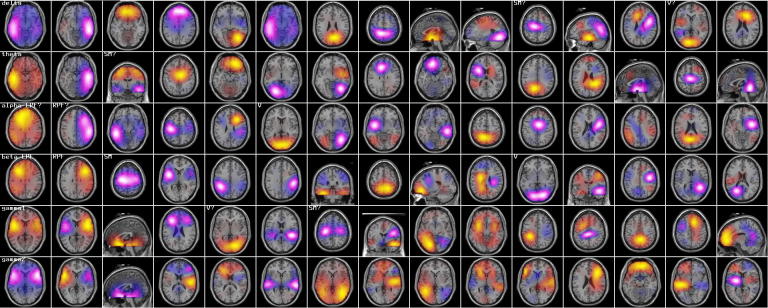
Overview of all 15 ICA components for each of the six frequency bands. A single slice is displayed in the plane providing the best overview of the activity foci in the respective component. The colours reflect positive (yellow/red) and negative (white/blue) areas of temporal correlation. Irrespective of direction, areas with the same colour are correlated, whereas areas of opposite colour are anti-correlated. Candidate components for each of the four networks of interest are indicated (SM = sensorimotor network, V = visual network, LPF/RPF = left/right parietofrontal network), as judged by visual interpretation. A question mark indicates questionable evidence. All four components can only clearly be identified in the beta band.

All four networks of interest (left and right parietofrontal, sensorimotor and visual networks) were clearly present as independent components in the beta range only (13–30 Hz) (Fig.1A, see Supplementary Fig. S1 for an overview of all components, and Fig. 2, bottom row, for axial slice views). Fig.1B shows the SDs per network and condition plotted over age. The parietofrontal networks appeared quite frontal-dominant, compared to previous findings (Brookes et al., 2011). However, frontal-weighted parietofrontal networks have been shown previously (Brookes et al., 2012), and these components were the most likely candidates to reflect these networks (Supplementary Fig. S1).

We performed two-way mixed-model ANOVAS on the SD within each network, with within-subjects measure Condition (eyes open/closed), and between-subjects measure Group (AD, EC, YC). For the left and right parietofrontal networks, these showed a significant main effect of Group (both *p* < 0.0003), but no main effect for Condition nor an interaction (all *p* > 0.1). Post-hoc t tests between groups (assessed at the *p* < 0.0125 Bonferroni-corrected level) showed the same highly significant pattern for both networks: AD < EC, EC > YC (all *p* < 0.0003) and no difference between YC and AD (*p* > 0.1). In the sensorimotor network, there was a weaker but still present main effect for Group (*p* = 0.0158) and a trend for Condition (*p* = 0.0890), but also an interaction (*p* = 0.0087). Post-hoc t tests showed that among the groups, the same pattern was present as in the left and right parietofrontal networks, where AD < EC and EC > YC (both *p* < 0.002), but no difference between YC and AD (*p* = 0.7744). Splitting the data by condition showed that the interaction reflects that this pattern remained significantly present in the eyes-open condition, but not for eyes-closed. In the visual network, there was a significant effect of Group only (*p* < 0.0140), although there was also a trend for Condition (*p* = 0.0799), but no interaction (*p* = 0.1940). Post-hoc t tests showed a different group pattern to the other three networks: AD < EC (*p* = 0.0054) and AD < YC (*p* = 0.0015) but no difference between EC and YC (*p* = 0.9636).

In summary, all networks except the visual network showed an increased mean SD of beta-band oscillatory amplitude with healthy ageing (EC > YC, Fig.1B). In contrast to a pattern of ‘exaggerated ageing’ in Alzheimer’s disease, all four networks showed a decreased mean SD for AD patients compared to healthy elderly controls. These effects were highly comparable in the eyes-open and eyes-closed states, especially in the parietofrontal networks.

There were differences in both average head position relative to the dewar (i.e., head angle or tilt) and beamformer weights between the AD and YC, and EC and YC groups. However, these were not significantly different between the AD and EC group, and thus could not explain our main network results pattern of AD < EC. Furthermore, there were three factors unequally distributed between the groups which may potentially bias the results: presence of grating, gender, and age. However, nuisance variable analyses showed that these three factors did not explain our main group effects. Details of these confound analyses are in the Supplementary Material; Appendix A.

Fig. 2 displays the four beta-band networks from the ICA when run separately for each group. Visual inspection suggests that the visual network appears most similar in each group, whereas the other networks appeared less symmetrical, more medially centered, or more diffuse for both EC and AD groups than in the YC group. Regardless of these differences, each network could be identified within the 15 components produced for each group, and peaks within each network were positioned in a highly similar location, suggesting a reasonable spatial consistency in this temporal ICA approach.

### 3.2 Whole-brain voxel-based analysis

**Fig. 3:**
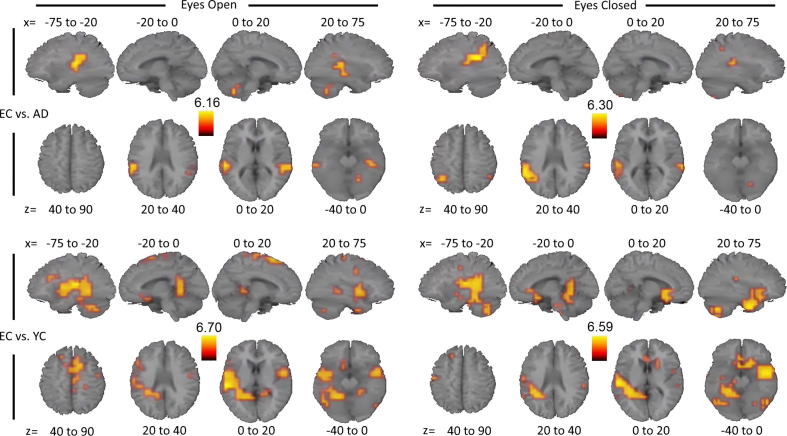
Voxel-based group comparisons of beta-band oscillatory envelope SD transformed to MNI space. Per comparison and eye state, images display four sagittal (top) and axial (bottom) slices, representing *t* values thresholded at voxel-corrected *p* < 0.05, averaged over the *x* and *z* Talairach coordinates as indicated (coordinates in mm). AD = Alzheimer’s disease patients, EC = Elderly controls, YC = Young controls. Orange/Yellow colours represent regions where EC had greater SDs than AD (upper panels), or than YC (lower panels). (For interpretation of the references to colour in this figure legend, the reader is referred to the web version of this article.)

To further clarify which brain areas contributed most profoundly to the partially overlapping ICA findings we performed a whole-brain voxel-based analysis. The results for the beta band are displayed in Fig. 3 and summarised in Table 1. Hotspots of significantly decreased beta-band envelope SD in AD compared to EC were localised to bilateral inferior parietal and superior temporal areas. Results in eyes-open and closed states were highly similar. For EC compared to YC, similar bilateral parieto-temporal areas were identified having greater SD in EC than YC, although they were more widespread and involved slightly more frontal areas. In all four comparisons, the left hemisphere had a greater and more extensive superior temporal focus than the right hemisphere.

**Table 1:** Whole-brain voxel-based analysis SD results listing clusters containing >1 voxel.

	EC vs. AD (Disease)		EC vs. YC (Ageing)	
	Eyes open	Eyes closed	Eyes open	Eyes closed
Delta (1–4 Hz) Theta (4–8 Hz)			Bilateral:	Bilateral:
			Parieto-temporal	Parieto-temporal
			Anterior cingulate	Anterior cingulate
			Posterior cingulate	Posterior cingulate
			Superior frontal	Superior frontal

Alpha (8–13 Hz)	Left:		Bilateral:	Bilateral:
	Middle-superior temporal		Middle temporal	Middle temporal
			Middle-superior frontal	Superior frontal
			Left:	Left:
			Superior-inferior temporal	Superior-inferior temporal
			Posterior cingulate	

Beta (13–30 Hz)	Bilateral:	Bilateral:	Bilateral:	Bilateral:
	Middle-superior temporal	Superior temporal	Inferior parietal	Inferior parietal
		Inferior parietal	Superior temporal	Superior temporal
			Pre- & postcentral gyri	Pre- & postcentral gyri
			Superior medial	Superior medial
			Parahippocampal gyri	Parahippocampal gyri
	Left:		Left:	Right:
	Inferior parietal		Middle frontal gyrus	Inferior temporal to inferior frontal

Gamma1 (30–50 Hz)			Bilateral:	Bilateral:
			Superior-middle frontal	Superior-middle frontal

Gamma2 (50–90 Hz)				Right:
				Precuneus^a^

a EC < YC, all other group differences were of the direction EC > AD and EC > YC, respectively.

**Supplementary Fig. S2:**
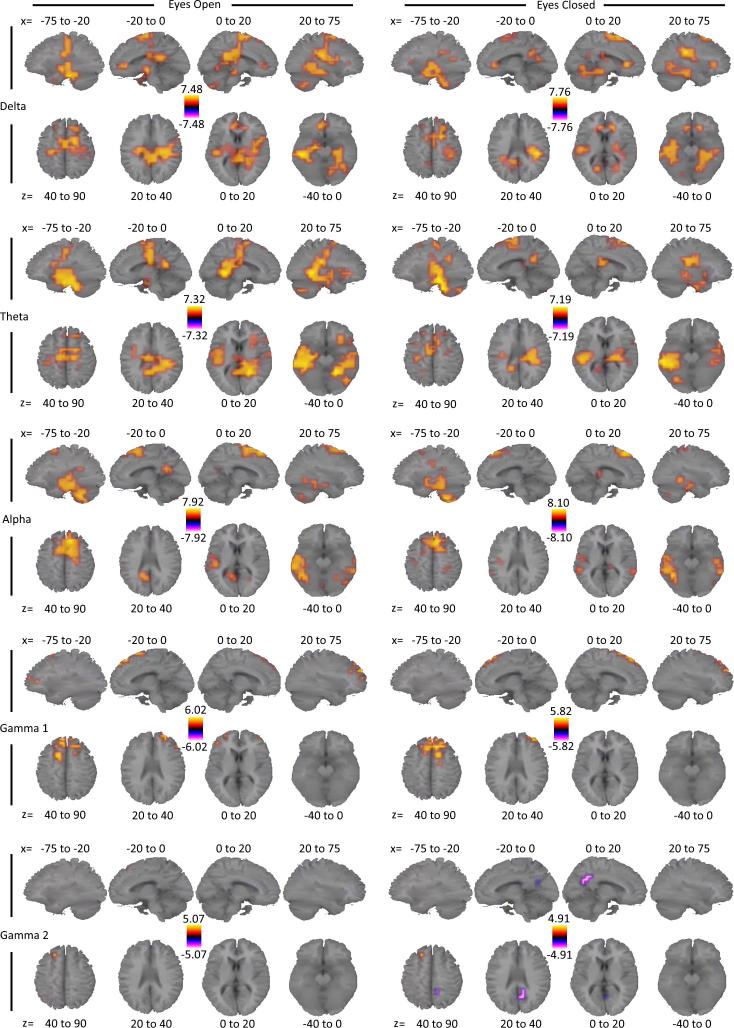
Voxel-based group comparisons of oscillatory envelope SD transformed to MNI space for the EC vs. YC comparison for all frequency bands, except the beta band, that showed significant differences. Per comparison and eye state, images display four sagittal (top) and axial (bottom) slices, representing *t* values thresholded at corrected *p* < 0.05 for correlation strength (the sum over nodes), averaged over the *x* and *z* Talairach coordinates as indicated (coordinates in mm). Gamma 1 = 30–50 Hz, Gamma 2 = 50–90 Hz. Orange/Yellow colours represent regions where EC had greater SDs than YC, blue/white colours represent regions where EC had lower SDs than YC.

Results for the remaining frequency bands are displayed in Supplementary Figs. S2 (EC vs. YC) and S3 (EC vs. AD) and Table 1. Within each band and group comparison, the eyes open and closed states generated very similar spatial patterns. Interestingly, the bilateral temporal areas showed increased SD for EC over YC similarly to the beta band in all bands below beta, with more extended clusters for the lower bands as well as a number of additional clusters. Importantly, the temporal clusters showing greater SD in EC compared to AD appeared to be unique to the beta band. There were very small clusters in left middle temporal cortex in both eye states of the alpha band, positioned inferior relative to the beta band, but this group comparison yielded no other consistent clusters in any other frequency band. None of the frequency bands showed a pattern that would support the accelerated ageing hypothesis (AD < EC < YC) for any voxels.

### 3.3 Node-based connectivity analysis

**Fig. 4:**
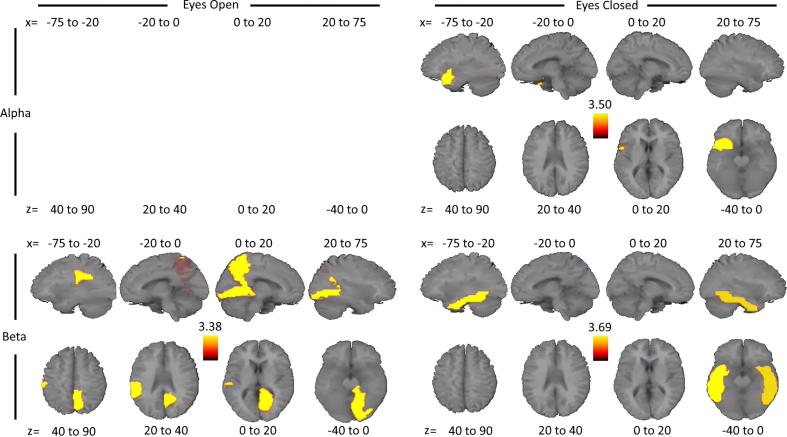
Node-based connectivity results for the EC vs. AD comparison. Orange/Yellow colours represent regions where EC had greater node strength than AD. Only group comparisons with significant differences are displayed. Each AAL atlas region represents the results for each respective node. Per comparison and eye state, images display four sagittal (top) and axial (bottom) slices, representing *t* values thresholded at corrected *p* < 0.05 for correlation strength (the sum over nodes), averaged over the *x* and *z* Talairach coordinates as indicated (coordinates in mm). (For interpretation of the references to colour in this figure legend, the reader is referred to the web version of this article.)

For OAC, significant group differences were present only in the alpha and beta bands (Fig. 4). In the alpha band, AD had reduced OAC strength compared to EC in the left superior temporal pole for eyes-closed only. In the beta band, this same reduced pattern was present in the right lingual gyrus and precuneus, and left supramarginal gyrus for the eyes-open state, and in the bilateral inferior temporal cortex during eyes-closed. For the EC versus YC comparison, no regions showed significant OAC strength differences for any frequency band.

In summary, consistency over eyes-open and closed states and across alpha and beta bands suggested that node strength based on amplitude correlations was reduced in AD patients compared to elderly controls mainly in bilateral temporal regions with a left-weighted bias. For PLI, there were no significant group differences for any frequency band in either eye state.

### 3.4 Partial least squares analysis on MMSE and other factors

Variables that showed a difference, or trend for a difference, between the EC and AD groups were: Years of education, Age, MMSE, Grating, Head motion, and Group.

For beta-band voxel-based SD, for left and right respectively, the PLS model explained 61.1% and 68.7% of the variance, which was significant for both sides (*p* < 0.0005). By pooling across models, the impact of the parameters could be assessed, which showed that for both sides, Group had the greatest impact (L: adjusted *R*^2^ = 0.32; R: 0.31; both *p* ≤ 0.0001). In addition to Group, MMSE also had a significant impact (L: adjusted *R*^2^ = 0.18; R: 0.19; both *p* < 0.002). The remaining variables (Years of education, Age, Grating and Head motion) did not have a significant impact on the models (all *p* > 0.1).

For AAL-OAC, the pattern of variable contributions was similar to that for voxel-based SD, although significance levels were reduced. For left and right respectively, the PLS model explained 34.3% and 37.7% of the variance, which was significant on the right (*p* = 0.034), but just under the significance level on the left side (*p* = 0.057). For both sides, Group had the greatest impact (L: adjusted *R*^2^ = 0.11; R: 0.15; both *p* < 0.02). In addition to Group, MMSE also had a significant impact (L: adjusted *R*^2^ = 0.10; R: 0.09; both *p* < 0.03). The remaining variables did not have a significant impact on the models (all *p* > 0.2).

In summary, MMSE scores explained a significant proportion of the variance in both amplitude measures at the peak group difference locations, in addition to group itself, whereas years of education, age, grating and head motion did not. This suggests that, although oscillatory amplitude variability and connectivity measures are primarily affected by disease, they are also sensitive to variability in cognitive integrity within each group.

### 3.5 Sensor-space analysis

We performed a sensor-space analysis, replicating the finding of decreased posterior individual alpha frequency (IAF) in AD compared to EC (Klimesch, 1999, Moretti et al., 2004, Lizio et al., 2011, Babiloni et al., 2015). We also aimed to replicate the commonly reported decrease in posterior alpha and beta power in healthy ageing with a further decrease in AD (Moretti et al., 2004, Osipova et al., 2005, Hsiao et al., 2013). Although this replication was not statistically significant, we did find that posterior beta-band suppression for eyes-open versus closed states was present in EC and YC, but not in AD (Supplementary Material; Appendix A).

To assess whether group differences in the sensor-space results between AD and EC were related to decreased cognitive functioning in AD, we correlated MMSE scores with the relevant neural measure within the AD group (*N* = 16). In case of a reduced value for AD compared to EC, a positive correlation with MMSE within the AD group would suggest that the reduction in AD compared to EC may be related to cognitive ability. We correlated posterior IAF, averaged over eye state, and posterior beta power, assessed separately for each eye state as there was an effect of beta suppression. Neither of these measures correlated with MMSE within the AD group (IAF: *R* = −0.120, *p* = 0.658; beta power eyes-open: *R* = 0.190, *p* = 0.480; beta power eyes-closed: *R* = 0.136, *p* = 0.615).

## 4 Discussion

Using whole-brain measures of local neural oscillatory activity and connectivity in the resting state we found that patients with Alzheimer’s disease (AD) present a distinct pattern of dysfunction across the brain compared to that evident in healthy ageing. AD-related differences were specifically localised to bilateral inferior parietal and superior temporal areas and were unique to the alpha and beta bands. In the beta band, a significant proportion of the variance in both oscillatory envelope variability and connectivity in areas of peak group difference was explained by cognitive integrity, in addition to group. Our results support the disconnection syndrome hypothesis and strongly suggest that AD shows distinct and unique patterns of disrupted neural functioning, rather than patterns of exaggerated healthy ageing, with disrupted local variability and cross-area connectivity playing a key role.

An important network of interest in AD is the default-mode network (DMN). Using a temporal ICA technique, we found the presence of four commonly known functional brain networks in the beta band. Network activity was increased in EC relative to YC in all networks except the visual network, and decreased in AD relative to EC in all four networks. We note that both parietofrontal networks were heavily frontally-dominant. Therefore, it is possible that our AD-related group difference in these networks was primarily driven by frontal areas. In our experience the DMN is not easily identified using the currently employed temporal MEG ICA technique, and it contains areas that lie relatively deep in the cortex, such as the posterior cingulate cortex (PCC), that are arguably not reliably measurable with MEG. Therefore, we did not directly investigate the DMN here. Nevertheless, the group differences in both regional area and cross-area amplitude connectivity identified regions that may be within the (posterior) DMN, or subnetworks of the DMN (Buckner et al., 2008), with further extension into the temporal lobes.

The current results fit in with the consensus over studies that central brain hubs, such as parietal and temporal areas, are most severely affected in AD (de Haan et al., 2012, Tijms et al., 2013, Stam, 2014), consistent with patterns of atrophy including a left-lateralised predominance (Baron et al., 2001, Karas et al., 2003). As the SD of oscillatory envelope amplitude can be interpreted as a measure of activity or variability (Muthukumaraswamy et al., 2013, Koelewijn et al., 2015), our voxel-based SD results are consistent with a reduced degree of fluctuation within synchronisation in the alpha and beta band in AD compared to EC (Stam et al., 2005), and additionally clarify localisation of this finding. One recent study also investigated beamformer-derived source space Hilbert envelope amplitude correlations, but obtained these for 8 regions in MCI and healthy elderly controls, and further obtained measures of white matter connectivity using diffusion imaging (Garcés et al., 2014). They found that both functional connectivity in the alpha band and structural connectivity were decreased for the MCI compared to EC group, most strongly so in inferior parietal areas. Considering that MCI is a likely precursor of AD, these findings in MCI further corroborate the present findings in AD, where some effect was also shown in the alpha band. MEG functional connectivity correlates well with structural connectivity overall, and this is strongest in the beta band and for areas of the DMN (Garcés et al., 2016). Therefore, our specific pattern of AD disruptions in DMN areas in the beta band compared to healthy ageing also fits well with diffusion studies in healthy ageing (Madden et al., 2009, Penke et al., 2010, Fjell et al., 2011) and AD (Acosta-Cabronero et al., 2010).

To our knowledge, our study is the first to conduct a whole-brain analysis of connectivity measures in MEG source space comparing AD to healthy ageing in multiple frequency bands. We performed this study in an attempt to exclude *a priori* decisions, which could either miss or enhance spatially or frequency-specific group effects. Engels et al. (Engels et al., 2016) very recently also conducted a whole-brain source-space analysis comparing an early-onset AD group to healthy elderly controls using a beamformer on eyes-closed only MEG resting-state recordings. Engels et al. found that 8–13 Hz peak frequency was reduced in AD, which strongly correlated with MMSE scores specifically in the hippocampus and right parietal areas. This specific localisation may explain why we did not find a correlation of IAF to MMSE scores as we calculated IAF over posterior sensors, rather than in source space, to allow for comparison to previous studies (Klimesch, 1999, Moretti et al., 2004, Lizio et al., 2011, Babiloni et al., 2015). We note that these previous sensor-space studies also did not find a correlation of IAF with MMSE (Moretti et al., 2004). Although IAF is consistently reported in AD, IAF may be a more generic marker of impairment of brain structure and/or function, as it is also reduced in traumatic brain injury patients, and correlates with cognitive preparedness in healthy individuals (Angelakis et al., 2004). The study by Engels et al. further found increased whole-brain lower frequency (mainly theta) power and decreased lower parieto-occipital alpha and frontal beta power, although power measures did not correlate with MMSE scores. Interestingly, Engels et al. did not find any differences localised to bilateral temporal areas.

Recent developments in studies using Positron Emission Tomography (PET) suggest that deposits of the tau protein, causing neurofibrillary tangles, are more closely related to cognitive performance and clinical symptoms, as well as patterns of atrophy, than amyloid-beta deposits (James et al., 2015, Brier et al., 2016, Ossenkoppele et al., 2016). It was long thought that amyloid-beta deposits were the most important cause of the neurodegeneration and cognitive and memory problems in AD. However, patterns of tau binding correlate better with both healthy ageing and AD than amyloid-beta (Brier et al., 2016, Ossenkoppele et al., 2016), and there is greater uptake of tau in ApoE4 carriers (carriers of the gene most strongly associated with the risk of developing AD) than non-carriers (Ossenkoppele et al., 2016). In AD patients, tau tracers bind specifically in the temporal lobes, including the hippocampus, whereas amyloid-beta tracers bind more diffusely in frontal and parietal cortices (James et al., 2015, Brier et al., 2016, Ossenkoppele et al., 2016). It is very interesting that these regions of specific tau protein deposits are in highly similar regions to the decreases in regional function and functional connectivity that we uniquely observed in parietotemporal cortices in AD compared to EC. We may therefore speculate that our current findings of oscillatory variability and connectivity in the alpha and beta bands relate to tau deposits, and/or atrophy, whereas the more diffuse patterns of peak frequency and low-frequency power (Engels et al., 2016) may be more related to amyloid-beta deposits. Future studies combining MEG and PET in the same subject sample could address this speculation.

In our results, local oscillatory activity and connectivity in the AD group were reduced compared to EC to the level of YC, which seems counter-intuitive. Results in mild AD and MCI activity often fall in between severe AD and healthy elderly controls (Koenig et al., 2005, Liu et al., 2014), suggesting a gradient from healthy ageing to AD in line with the severity of cognitive symptoms. However, not all findings are concordant with this gradient. Our results fit partially with an fMRI study in healthy ageing, where increases in BOLD activity were found within the sensorimotor and right parietofrontal networks, although decreases were found in the DMN and visual networks (Mowinckel et al., 2012). Activity within the DMN is typically found to be reduced in AD compared to EC (Greicius et al., 2004, Liu et al., 2014), in line with our present findings. However, van Dam et al. (Van Dam et al., 2013) found that activation in MCI was greater than in EC in parietotemporal areas, the PCC, and other regions of the DMN. Furthermore, Fillipini et al. (Filippini et al., 2009) found that ApoE4 carriers had increased activity compared to non-carriers in parts of the DMN, as well as in the sensorimotor network, but not in the visual network. This finding also does not fit with the predictions from the intuitive ‘accelerated ageing’ gradient, suggesting that an opposite pattern between changes in healthy ageing or precursor stages of AD and AD itself occurs across different modalities and is not an unusual finding.

We aimed to perform an unbiased whole-brain analysis avoiding *a priori* decisions as much as possible. Nevertheless, our study has a number of limitations. First, we are comparing small groups with potential differences other than age and AD diagnosis. Especially comparing the healthy young and elderly groups needs some caution as, in addition to age, they differed to a degree in the amount of head motion and position, and possibly in other factors that we did not measure, such as muscle activity, atrophy or ventricle shape and size. It remains to be assessed what the effects are of changes in ongoing head motion or position on oscillatory neural signals during the resting-state, as this is known to cause artefactual correlation and confounds in BOLD resting-state networks in fMRI (Mowinckel et al., 2012, Murphy et al., 2013). Importantly, here, the difference between AD and EC could not be explained by factors such as head position, motion, or a slight difference in age. Furthermore, as the patterns of our results were in the opposite direction between EC and YC to what they were between AD and EC, we can at least interpret our differences between AD and EC as not being due to a pattern of accelerated ageing and rather reflecting unique and specific patterns of dysfunction.

Second, in an attempt to provide a global, comprehensive, and un-biased study we provide only a selection of many possible analysis measures in order to at the same time restrict multiple comparisons. We chose to only calculate the strength of amplitude and phase correlations over nodes. The resulting measure reflects the degree of ‘importance’ of the node in the network. However, using strength, we lose specific area-to-area connectivity information. If specific connections are more important in AD than specific areas, this may explain why we did not find any group differences in the phase lag index. Fronto-parietal and fronto-temporal connections appear to be most affected in AD (Babiloni et al., 2016). Perhaps the parietotemporal areas that had reduced amplitude strength in AD compared to EC connect to frontal areas specifically. Furthermore, there are many different and additional ways to calculate correlation among and across oscillatory neural activity, and many graph theoretical measures that can be applied to address different questions regarding connectivity and network behaviour. These will need to be carefully applied to address specific hypotheses which were beyond the scope of this study, and have the potential to further unravel the complexity of network activity and organisation in AD and healthy ageing.

Third, we also did not divide our alpha band into an upper and lower band as has been suggested to be best practice in AD research (Moretti et al., 2004, Cantero et al., 2009), although three smaller bands have also been suggested (Klimesch, 1999). However, we made this decision to limit the number of comparisons. Our main findings were strongest in the beta band, but it is possible that our findings may have extended into one of these alpha sub-bands and are not evident in our broader alpha band. Finally, our AD group was heterogeneous in terms of medication. Unfortunately, our small AD group sample prevented investigation of the effect of medication.

## 5 Conclusion

In summary, in an unbiased, whole-brain assessment of oscillatory brain functioning, we found converging results from two analyses suggesting that both local and network oscillatory activity is abnormal in AD. Our results suggest that bilateral parietotemporal areas are selectively affected in the alpha and beta bands. We found that these patterns, especially amplitude connectivity, were specific to AD compared to healthy ageing, and both beta-band amplitude measures were partially explained by cognitive integrity. Additionally, we investigated an open question in the literature as to what the effect was of recording resting-state keeping eyes closed versus open, and found that these resulted in highly similar patterns of group differences. Based on similarity to recent PET findings, we speculate that the functional abnormality in parietotemporal areas may be related to neurodegeneration due to the accumulation of tau rather than amyloid-beta, although this will need further investigation. As MEG is easily tolerable by AD patients, and is less susceptible to contra-indications, compared to more invasive, noisy and constraining procedures such as PET and MRI, our findings may contribute towards developing an easy, non-invasive tool for a disease biomarker for AD.

## Figures and Tables

**Supplementary Fig. S3:**
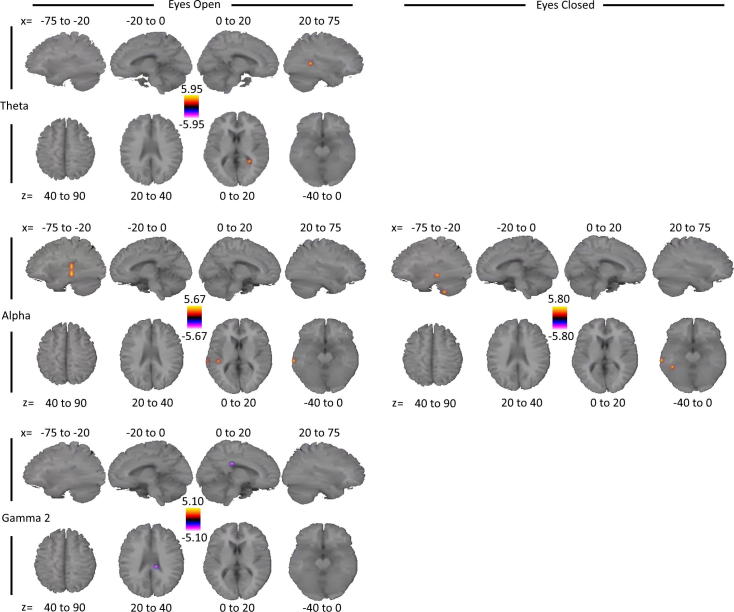
Voxel-based group comparisons of oscillatory envelope SD transformed to MNI space for the EC vs. AD comparison for all frequency bands, except the beta band, that showed significant differences. Per comparison and eye state, images display four sagittal (top) and axial (bottom) slices, representing *t* values thresholded at corrected *p* < 0.05 for correlation strength (the sum over nodes), averaged over the *x* and *z* Talairach coordinates as indicated (coordinates in mm). Gamma 2 = 50–90 Hz. Orange/Yellow colours represent regions where EC had greater SDs than AD, blue/white colours represent regions where EC had lower SD than AD.
