# Supplementary material for: Alzheimer's disease disrupts alpha and beta-band resting-state oscillatory network connectivity
Source: Clin Neurophysiol. 2017 Nov;128(11):2347–57. doi: 10.1016/j.clinph.2017.04.018 (PMC5674981; doi:10.1016/j.clinph.2017.04.018)
Supplement: Supplementary data 1 [file mmc1.docx]

# Supplementary material

# Appendix A.

## Sensor-space analysis

### Materials and methods

For the sensor-space analysis, we imported the pre-processed, filtered, epoched and split datasets, identical to those entered into the ICA analysis, into MATLAB using the FieldTrip toolbox (Oostenveld et al. 2011). Our radial gradiometer data were first converted into planar gradiometer geometry to better interpret the location of sources of magnetic signal flow at the scalp. We then performed a time-frequency analysis on the planar sensor data extracting the power spectrum at every sensor within 1-100 Hz using the 'multitaper method' based on a hanning window. To obtain posterior alpha peak frequency, we averaged the power spectrum over all occipital sensors, and calculated the frequency at maximum power between 5-15 Hz (‘broad-band’ alpha to capture peaks at the edges of the band). For alpha and beta power, we obtained the mean power over the same occipital sensors between 8-13 Hz (alpha) and 13-30 Hz (beta), respectively.

### Results

We successfully replicated the common finding of a posterior Individual Alpha Frequency (IAF) shift in Alzheimer’s Disease and additionally investigated the effect of eye ‘state’ (Condition: open or closed) (Supplementary Figure A.1 A). A two-way mixed model ANOVA showed a significant main effect of Group (F(2,55)=14.75, p<0.001), but not Condition (F(1,55)=0.20, p=0.656), and a significant interaction between the two factors (F(2,55)=6.85, p=0.002). Planned pairwise comparisons revealed that overall (at p=0.05, Bonferroni corrected), IAF was significantly different for AD (mean 7.97 Hz) < EC (mean 10.12 Hz) as well as AD < YC (mean 10.19 Hz), but there was no significant difference between EC and YC, supporting our hypothesis. Furthermore, in the eyes-open condition, this same pattern was significant. In the eyes-closed condition, IAF was lower in AD than both other groups, but there was an additional significant difference between EC and YC, where group average IAF was higher in YC than in EC.

We next tested the common hypothesis that posterior alpha and beta power are reduced in EC compared to YC, and further reduced in AD compared to EC (Supplementary Figure A.1 B, C, D). Mean posterior alpha power showed a significant effect of Condition (F(1,55)=23.23, p<0.001), but not Group (F(2,55)=1.18, p=0.315), nor an interaction (F(2,55)=1.53, p=0.266). Thus, our hypothesis was not supported. However, we did see evidence for the commonly known alpha suppression (strongly reduced alpha power when eyes are open compared to closed). The results were highly similar when mean posterior alpha power was calculated using a 5 Hz band around IAF, as suggested to be best practice (Klimesch, 1999). We found a similar pattern of results for posterior beta power. The effect of Condition was significant (F(1,55)=23.35, p<0.001), showing beta suppression; there was no significant effect of Group (F(2,55)=1.00, p=0.376), but there was also a significant interaction (F(2,55)=3.22, p=0.048). Pairwise comparisons showed that both the EC and YC group had significant eyes-open beta suppression (p<0.05, Bonferroni corrected), but the AD group did not.

## Supplementary Figure A.1.


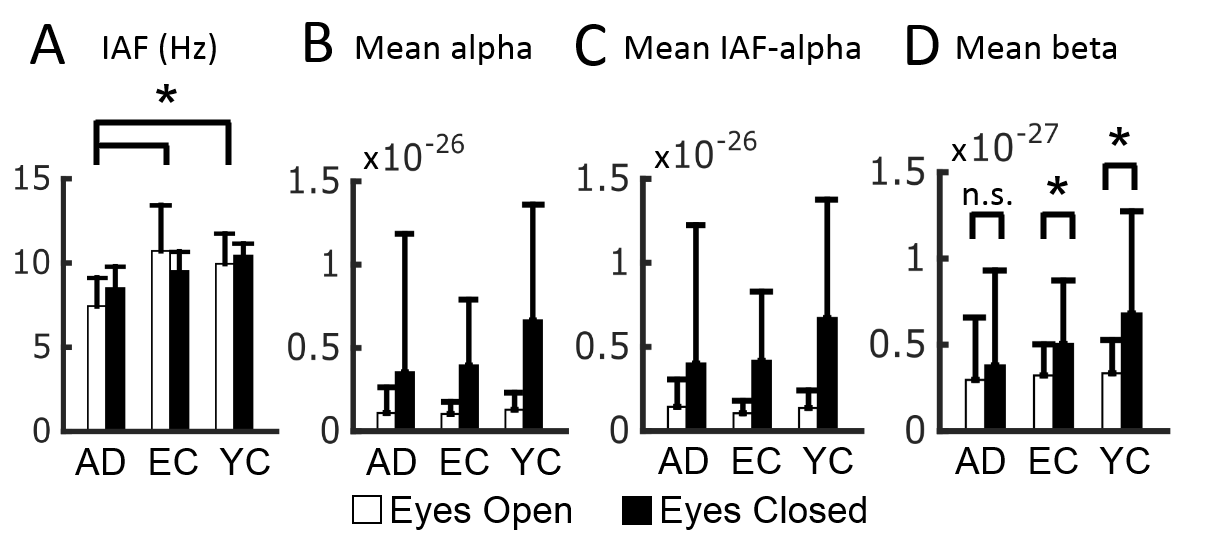


*Supplementary Figure A.1. Sensor space results (posterior sensors average). A) Individual Alpha Frequency (IAF) obtained between 5-15 Hz. B) Mean alpha power taken between 8-13 Hz. C) Mean alpha power taken for 5 Hz around IAF. D) Mean beta power taken between 13-30 Hz. Error bars represent SDs of group mean. *p<0.05, Bonferroni corrected. n.s. = not significant (p>0.05).*

## Correlations ICA and sensor-space findings

We present novel findings in beta-band oscillatory resting-state network variability. We have further replicated common findings in the literature, in particular a shift in IAF in AD compared to healthy ageing, and additionally found reduced eyes-open beta power suppression in AD. The patterns of results in the sensor-space analysis (AD < EC, but no difference EC and YC) were different than the pattern of results in the ICA network analysis (AD < EC and EC > YC). Nevertheless, the patterns for AD disease state (AD vs. EC) were in the same direction. To understand whether the two measures convey similar information regarding disease state, we investigated whether IAF correlated with beta-band network SD over the whole group (N=58). We averaged measures over eyes-open and closed states where there was no effect of Condition (IAF, all four networks SDs). In addition, eyes-open beta power suppression differed over the groups. We therefore also correlated the network SDs to eyes-open and closed mean posterior beta power. Thus, we correlated 4 network values to 3 sensor-space values, yielding 12 correlations. We therefore assessed effects at a Bonferroni-corrected threshold of p<0.0042.

There were no significant correlations for any of the parietofrontal and sensorimotor networks (all p>0.1). For the visual network, there were a few trends with correlation to sensor-space measures, though none of the correlation p values were below the Bonferroni corrected threshold (visual network SD to mean IAF (R=0.315, p=0.016), to eyes-open mean beta power (R=0.339, p=0.009), and to eyes-closed mean beta power (R=0.315, p=0.016)).

## Resting-state independent component network analysis: head position, beamformer weights and nuisance variables

A difference in head position (i.e., angle or tilt) between the groups could potentially influence the ICA results. We assessed whether there were any differences between the groups by inspecting the measured head-to-dewar distances for each coordinate of each fiducial. One-way ANOVAs on each of these measures, with post-hoc comparisons for significant ANOVAs, revealed that a number of fiducial coordinates were significantly different between groups (nasion Z coordinate (p=0.04), right pre-auricular X and Z, left pre-auricular Y and Z, all p<0.02). Post-hoc comparisons on these coordinates showed that any differences present were only significant (p<0.05) between EC and YC or between AD and YC, whereas there was no difference between AD and EC for any fiducial or coordinate. Most of the differences between groups were for the left and right pre-auricular fiducials in the axial dimension, suggesting a superior/inferior shift. This could either imply that elderly participants (both AD and EC) gradually slumped in the MEG chair to a different degree than younger participants, or it could imply a difference in head/neck posture, which would be in line with ‘forward head posture’ often developed in older age. Importantly, as there were no differences in head position between AD and EC, a tilted head position bias cannot explain our main network results pattern of EC>AD.

There were significant differences in beamformer weights between the groups. However, the pattern of results could not be explained by a bias in beamformer weights. Two-way mixed ANOVAs for each network, followed by post-hoc pairwise comparisons, showed a significant main effect of Condition for all four networks (all p<0.001), where all weights were of a greater magnitude in the eyes-closed than eyes-open condition. There also was a main effect of Group for the left and right parietofrontal and sensorimotor networks (p<0.002), but not for the visual network (p=0.605). Furthermore, there was a significant interaction between Group and Condition for the right parietofrontal (p=0.025) and visual networks (p=0.006). Post-hoc paired comparisons showed that for the left and right parietofrontal and sensorimotor networks, significant Group differences were EC>YC (all p<0.001), and AD>YC (all p<0.040), but not between AD and EC (all p>0.177, even when uncorrected). These differences in the square root of weights magnitude may reflect differences between elderly and young groups related to head motion or position, but clearly cannot explain the oscillatory network effects of AD<EC, and the absence of a difference between eyes open and closed states in three of the four networks.

There were three factors unequally distributed between the groups which may potentially bias the results: presence of grating, gender, and age. We repeated the two-way mixed model ANOVAs on network SD, with Bonferroni corrected post-hoc tests, using each of these three factors as a nuisance variable in turn. Neither Grating nor Gender affected our main results for the parietofrontal and visual networks. For the sensorimotor network, including either Grating or Gender as a nuisance factor generated a Condition * Group interaction in addition to the Group effect, where post-hoc tests showed that the group differences were significant in the eyes-open condition only. However, the Grating or Gender factor itself was not significant in any of these comparisons (all p>0.4). Finally, including Age as a nuisance variable affected the ANOVAs for the parietofrontal networks (Age factor: p=0.018 left; p=0.032 right), but the effect of Group was still significant for both networks. Predictably, Age removed the difference between EC and YC, but importantly, AD<EC was still significant. The factor Age was not significant for the sensorimotor or visual networks. In the sensorimotor network, however, including Age reduced the effect of Group to a trend (p=0.057), whereas the main results of the ANOVA for the visual network were not affected.

## Appendix References

Klimesch W. EEG alpha and theta oscillations reflect cognitive and memory performance: a review and analysis. Brain Res Brain Res Rev. 1999;29:169–95.

Oostenveld R, Fries P, Maris E, Schoffelen JM. FieldTrip: open source software for advanced analysis of MEG, EEG, and invasive electrophysiological data. Comput Intell Neurosci. 2011;2011;156869.
